# The Regulation of ROS- and BECN1-Mediated Autophagy by Human Telomerase Reverse Transcriptase in Glioblastoma

**DOI:** 10.1155/2021/6636510

**Published:** 2021-03-08

**Authors:** Xuelu Ding, Ziyang Nie, Zhaoyuan She, Xue Bai, Qiuhui Yang, Feng Wang, Fei Wang, Xin Geng

**Affiliations:** ^1^Department of Biochemistry and Molecular Biology, School of Basic Medical Sciences, Tianjin Medical University, Tianjin 300070, China; ^2^Key Laboratory of Immune Microenvironment and Disease (Ministry of Education), Tianjin Medical University, Tianjin 300070, China; ^3^Department of Neurology, Tianjin Neurological Institute, Tianjin Medical University, General Hospital, Tianjin 300052, China; ^4^Department of Genetics, School of Basic Medical Sciences, Tianjin Medical University, Tianjin 300070, China; ^5^Department of Neurology, General Hospital, Tianjin Medical University, Tianjin 300052, China

## Abstract

Glioblastoma (GBM) is the most common and aggressive malignant brain tumor with high morbidity and mortality. Human telomerase reverse transcriptase (hTERT), the catalytic subunit of human telomerase, is overexpressed in most cancers including GBM. It is well known that hTERT can compensate telomere shortening to immortalize cells. However, in addition to the canonical function, hTERT has the roles beyond canonical telomere maintenance. To further understand the effects of hTERT on glioblastoma progression, we investigated the role of hTERT in regulating autophagy—a conserved pathway, by which cells deliver cellular organic material and impaired organelles to the lysosomes for degradation and recycle these cargos to produce energy under a stressful condition. Our results showed that downregulation of hTERT impaired autophagy levels by suppressing BECN1/beclin-1 and induced an increase of reactive oxygen species (ROS), which resulted in cell death ultimately. On the contrary, overexpression of BECN1 or treating cells with the antioxidant N-acetylcysteine (NAC) could restore the survival of hTERT knockdown cells. Our study will provide an additional basis of telomerase-targeting therapy for future clinical anticancer treatment.

## 1. Introduction

Glioblastoma (GBM) is classified as grade IV glioma by the World Health Organization and accounts for about 20% of all brain tumors. From a global perspective, there are 0.59~3.69 cases out of 100,000 people [1]. Despite the improvement of medical technology and the rigorous treatment that patients received, it is still difficult to remove GBM tumors by surgical resection due to their invasion to the surrounding tissues. As a result, recurrence often occurs in GBM patients, and the median survival is less than two years after diagnosis [2–4]. According to the 2016 WHO classification, increasing evidence demonstrated the genetic heterogeneity of GBM [5]. To date, up to 45 genes were found to contain mutations, including *IDH1/IDH2*, *TP53*, *ATRX*, *TERT*, *NF1*, *PTEN*, and *EGFR* [6]. Thus, even within a single glioblastoma, a combination of treatments may be required for different cell subtypes, greatly exacerbating the difficulty of treating this type of tumor.

Telomerase is an RNA-dependent DNA polymerase, which can add TTAGGG repetitive sequences to telomeres via de novo DNA synthesis, thus enabling cell proliferation and immortalization [7]. Telomerase is composed of two main subunits: telomerase reverse transcriptase (TERT) and an RNA component (TERC). TERT is the core catalytic subunit of telomerase holoenzyme, and the gene expression level is crucial to telomerase activity [8]. In normal somatic cells, telomerase activity is difficult to detect, while most cancer cells reactivate telomerase thus can proliferate unlimitedly [9]. In GBM, researchers found frequent mutations in TERT promoter, which are considered a mechanism of increasing TERT expression [10, 11]. Therefore, TERT may be involved in the progression and used for analyzing prognosis of glioblastoma. It is well studied that telomerase can lengthen telomeres to maintain the stability of chromatin structure. However, emerging evidence shows the extratelomeric roles of TERT, including mitochondria fitness regulation, antiapoptosis, and response to DNA break repair [12–14]. Furthermore, by interacting with transcription factors or chromatin modulating factor, such as MYC, NF-*κ*B, and BRG1, the gene expression of TERT can be regulated independently of telomerase activity [15–17]. Hence, it is necessary to explore new features of TERT.

Autophagy is an evolutionally conserved process of degradation, by which double-membraned vesicles (autophagosomes) deliver intracellular components that need to be fused with lysosomes where the materials are resolved and recycled into the cytosol [18]. Basal autophagy maintains the stability of the intracellular environment via turnover of dysfunctional proteins and organelles [19]. However, when cells are under a stressful state (for example, nutrient deprivation), autophagy is strongly upregulated and provides a survival mechanism by promoting the circulation of nutrients, preventing the accumulation of misfolded proteins, decreasing reactive oxygen species (ROS), maintaining the function of organelles, and regulating intracellular signaling pathways [20]. In recent years, there has been increasing evidence in the role of oxygen free radicals in tumorigenesis and the dual function of antioxidants in the prevention and treatment of cancer [21, 22]. In comparison with healthy cells, cancer cells exhibit increased levels of ROS. This alteration of cellular redox homeostasis may have a series of effects on cancer cells, influencing cell proliferation, invasion, metastasis, and sensitivity to anticancer drugs. The altered oxidant/antioxidant balance, resulting from increased production of ROS, inactivation and decrease of the antioxidant enzymes, can induce oxidative damage and lipid peroxidation processes leading to cell death [22–24].

In the present study, we explore to demonstrate the regulation of ROS and autophagy by TERT and the subsequent effects on GBM survival, aiming to provide a more molecular basis for TERT-targeting therapies. We investigated the role of TERT in regulating ROS and autophagy, and the results showed that downregulation of hTERT can impair autophagy levels by suppressing BECN1 and lead to an increase of ROS, which resulted in cell death ultimately. On the contrary, overexpression of BECN1 or treating cells with the antioxidant N-acetylcysteine (NAC) could restore the survival of TERT knockdown cells. Our study will provide an additional basis of telomerase-targeting therapy for future clinical anticancer treatment.

## 2. Materials and Methods

### 2.1. Databases for Bioinformatics

The expression levels of hTERT in normal tissues and cancer tissues were searched in GEPIA database (http://gepia.cancer-pku.cn/) and CGGA database (http://www.cgga.org.cn/). The correlation of the expression levels of hTERT and patients' survival time in glioblastoma samples and normal samples were searched by TCGA database (http://portal.gdc.cancer.gov), and the statistical significance was analyzed by the SPSS software. The STRING database (http://string-db.org/) was used to query the interacting proteins of the TERT and BECN1 proteins. The STRING database can be used to annotate the structure, function, and evolutionary properties of proteins. It can also explore and predict protein interaction networks, provide new research directions for future experiments, and provide efficient mapping of cross-species predictions.

### 2.2. Cell Culture

The human glioblastoma cell line U87 was generously gifted by Professor Xudong Wu from Tianjin Medical University, originated from ATCC. Cells were cultured in Dulbecco's Modified Eagle's Medium (DMEM; Biological Industries), supplemented with 10% fetal bovine serum (FBS; Biological Industries) in a humidified chamber with 5% CO_2_ at 37°C. To establish stable TERT knockdown cell line, lentiviruses carrying TERT-targeting shRNA (GenePharma) were transduced into U87 cells along with 5 *μ*g/mL polybrene (GenePharma), and cells were sorted by 1 *μ*g/mL puromycin (Thermo Fisher). Plasmid transfection was carried out using Lipofectamine 3000 (Invitrogen), as recommended by the manufacturer. We also used the ROS scavenger, N-acetylcysteine (NAC; Beyotime Biotechnology), at a concentration of 3 mM.

### 2.3. Plasmid

The *BECN1* gene was amplified by PCR from the cDNA of U87 cells. The PCR products were purified with a Gel Extraction Kit (TIANGEN). Then, restriction endonucleases were used to digest the purified products and pLVX-IRES-puro vectors. After purification, they were ligated by T4 DNA ligase (Takara). Finally, the plasmids were amplificated in DH5*α E. coli* cells and identified by sequencing.

### 2.4. Quantitative Real-Time PCR (qPCR)

Total RNA was isolated using the Total RNA extraction kit (TIANGEN) according to the manufacturer's instructions. 1 *μ*g RNA was used as a template to synthesize cDNA using the GoScript Reverse Transcription System (Promega). All qPCRs were carried out using the Roche LightCycler System with SYBR green incorporation (Roche). The primers used in this study are from primer bank (https://pga.mgh.harvard.edu/primerbank/index.html) and are as follows: GAPDH, 5′-GGAGCGAGATCCCTCCAAAAT-3′ and 5′-GGCTGTTGTCATACTTCTCATGG-3′; TERT, 5′-CCGATTGTGAACATGGACTACG-3′ and 5′-CACGCTGAACAGTGCCTTC-3′. Each sample was assayed in triplicate. The relative mRNA level was determined using the 2^−*ΔΔ*Ct^ method.

### 2.5. Western Blotting

Cells were lysed in RIPA lysis buffer supplemented with phosphatase inhibitor cocktail and protease inhibitor cocktail (Roche). Protein content was measured with the BCA Protein Assay (Thermo Fisher Scientific). Equal amounts of protein extracts were separated on the SDS-PAGE gel, followed by electrotransfer onto a PVDF membrane (Millipore). The blots were subsequently incubated for 2 h in blocking buffer (5% nonfat milk in TBST). The membranes were incubated in 4°C overnight with monoclonal antibodies and followed by corresponding secondary anti-rabbit or anti-mouse antibodies for 2 h. The antibodies used for Western blot were as follows: Beta-actin (ProteinTech), TERT (ABclonal), mTOR (ABclonal), p-mTOR (Ser2448, Cell Signaling Technology), BENC1 (Santa Cruz Biotechnology), p62 (Cell Signaling Technology), LC3 (Abcam), and secondary anti-rabbit or anti-mouse antibodies (ProteinTech). The protein was detected with the ECL Blotting Detection Reagents (Thermo Fisher Scientific).

### 2.6. Telomere Repeat Amplification Protocol (TRAP) for Telomerase Activity

Telomerase activity of U87 cell was assayed with telomeric repeat amplification protocol (TRAP) performed as previously described. Briefly, after washing with PBS, 10^6^ cells were resuspended in 500 *μ*L 1× CHAPS lysis buffer, incubated on ice for 30 min, and then centrifuged at 13,000 g at 4°C for 30 min. Then, 1 *μ*L was used for the PCR reaction. PCR products were separated on 12% nondenaturing polyacrylamide gels and then stained with SYBR Green I for 30 min, visualized by UVP imaging system. Fluorescence density was quantified by ImageJ.

### 2.7. Cell Proliferation Assay with CCK-8

Cells were counted and seeded in 96-well plates. The Cell Counting Kit-8 (CCK-8) (Beyotime Institute of Biotechnology) was used to determine the cell proliferation rate according to the manufacturer's instructions. Briefly, after the cells grew for 0, 24, 48, and 72 h, respectively, they were incubated in CCK-8 reagent at 37°C for 2 h. Then, absorbance at 450 nm was measured using a microplate reader.

### 2.8. Measurement of Cellular ROS

The intracellular ROS level was determined using DCFH-DA Reagent (Beyotime Institute of Biotechnology) following the manufacturer's instructions. Briefly, cells were incubated with DCFH-DA at 37°C for 20 min. Then, cells were washed with serum-free medium for 3 times. The fluorescence intensity of DCFH-DA was detected using a fluorescence microscope quantified by ImageJ or analyzed by flow cytometer.

### 2.9. Cell Viability Assessment

Cell viability measurements were conducted using CCK8 (Beyotime Institute of Biotechnology) a4ccording to the manufacturer's instructions. Briefly, the prepared cells grown in 96-well plates were treated with 10 *μ*L of CCK-8 reagent after standard culture for 24 h. Next, cells were incubated at 37°C for 2 h. Finally, the absorbance at the wavelength of 450 nm was measured by a microplate reader.

### 2.10. Statistical Analysis

Data were analyzed by one-way ANOVA with Fisher's LSD test as indicated and shown as mean ± SEM from at least three independent experiments unless otherwise indicated. Data analysis was carried out using GraphPad Prism5.0 (GraphPad Software). *P* values of < 0.05 were considered statistically significant.

## 3. Results

### 3.1. TERT Is Overexpressed in GBM and Affects the Survival Rate of GBM Patients

**Figure 1:**
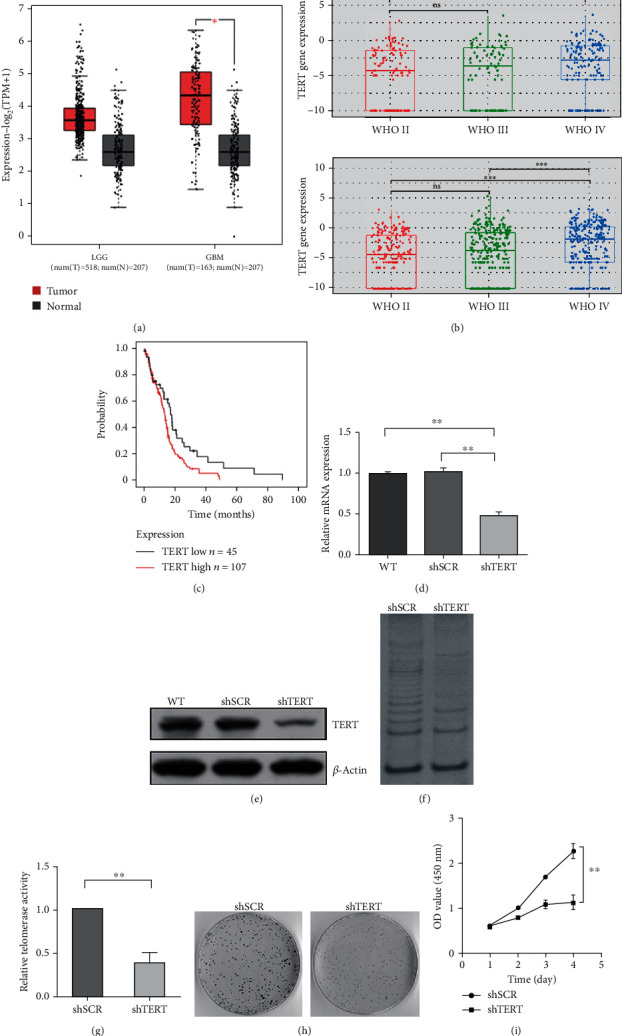
Bioinformatics analysis of hTERT in GBM and the influence of hTERT on telomerase activity, cell growth, and proliferation. (a) Box plot of LGG (low-grade glioma), GBM (glioblastoma) patients, and the corresponding normal controls from the GEPIA dataset. (b) Box plot of TERT gene expression in samples of Chinese glioma patients from CGGA dataset (above: CGGA mRNA-seq325: num (II) = 109, num (III) = 85, and num (IV) = 146; below: CGGA mRNA-seq693: num (II) = 194, num (III) = 261, and num (IV) = 256). (c) Survival analysis of GBM patients with high TERT levels and low TERT levels from TCGA dataset (TERT low: *n* = 45; TERT high: *n* = 107). (d) TERT mRNA levels and (e) protein levels in TERT knockdown cells and control cells. (f), (g) Telomerase activity assayed by TRAP. (h) Cell survival rate by clone formation experiment. (i) Growth curve of TERT knockdown cells and control cells by CCK-8. ∗∗*p* < 0.01 and ∗*p* < 0.05 were considered statistically significant.

By searching the GEPIA database, we found that hTERT mRNA levels were higher in low-grade gliomas (mainly including Grades II and III glioma) and glioblastoma (Grade IV glioma) than their corresponding normal tissues. And the difference between the tumor samples of GBM and the relative normal samples was statistically significant (Figure 1(a)). The expression levels of hTERT mRNA also increased from low malignancy to high malignancy in samples from Chinese GBM patients. Besides, the difference between samples of Grade II and Grade IV was statistically significant (Figure 1(b)). To further identify the importance of TERT in GBM, we also analyzed the relationship between TERT expression and survival time of GBM patients using datasets from TCGA. The results showed that GBM patients with lower expression levels lived longer than those with higher levels of TERT (Figure 1(c)). These results indicated that elevated expression of TERT may play a tumor promoting role during GBM progression.

### 3.2. Downregulation of TERT Impairs Cell Survival and Proliferation

To seek for the effects of TERT expression on cell proliferation of GBM, we transfected U87 cell lines with lentiviruses carrying TERT-targeting small hairpin RNA and constructed a stable cell line in which TERT expression was downregulated. After 48 h of transfection, we measured the silencing efficacy of TERT by qPCR and western blotting. The molecular weight of TERT protein detected was 127 kDa. The results showed that compared to control cells, the level of TERT expression was decreased by about 50% in TERT knockdown cells (Figure 1(d) and Figure 1(e)). The telomerase activity of TERT knockdown cells was significantly decreased assayed by TRAP (Figures 1(f) and Figure 1(g)). Besides, by cell clone formation experiment and cell proliferation detection, we found that TERT downregulation significantly suppressed cell survival rate (Figures 1(h) and 1(i)), indicating that TERT plays an important role in the growth and proliferation of GBM cells.

### 3.3. Autophagy and ROS Participate in the Regulation of GBM Growth and Proliferation by hTERT

**Figure 2:**
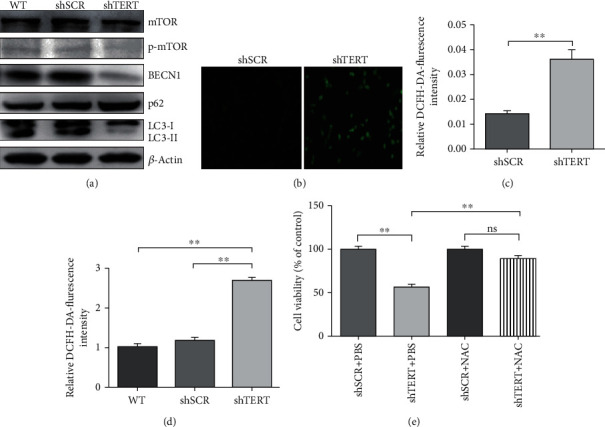
Autophagy and ROS participate in the regulation of GBM growth and proliferation by hTERT. (a) Western blotting images of autophagy-related proteins from TERT knockdown cells and control cells. (b, c) Graphical representation of ROS levels in TERT knockdown cells and control cells analyzed by fluorescence microscope. (d) Graphical representation of ROS levels in TERT knockdown cells and control cells analyzed by flow cytometer. (e) Cell viability of TERT knockdown cells treated with antioxidant NAC and PBS as a control. ∗∗*p* < 0.01 and *p* < 0.05 were considered statistically significant.

Autophagy is an essential mechanism for cell homeostasis. When cells are subjected to various stress states, autophagy will be upregulated to promote cellular and mammalian survival. To explore the effects of TERT knockdown on autophagy in GBM, we detected the expression levels of some major molecular markers of autophagy, mTOR, p-mTOR, BECN1, p62, and LC3B by western blotting. As shown in Figure 2(a), TERT knockdown was found to decrease the expression of BECN1, the conversion of LC3B from LC3B-I to LC3B-II, and increase the expression of p62, indicating that TERT deficiency suppresses the process of autophagy in U87 cells. Interestingly, the expression level of p-mTOR did not significantly increase after TERT knockdown. Comparatively, the change of BECN1 expression level was more significant than that of p-mTOR. Therefore, we speculated that BECN1 mediated the suppression of autophagy by TERT knockdown.

ROS are usually small and short-lived molecules, whose increase may lead to oxidative stress [22]. In comparison with healthy cells, cancer cells exhibit increased levels of ROS. This alteration of cellular redox homeostasis may have a series of effects on cancer cells, influencing cell proliferation, invasion, metastasis, and sensitivity to anticancer drugs. It is commonly accepted that increased level of ROS induces autophagy, which serves to decrease oxidative damage [25]. Here, we wonder that if intracellular ROS levels will accordingly rise after the inhibition of autophagy by TERT knockdown. Therefore, we detected cellular ROS levels using DCFH-DA probes and analyzed the results using fluorescence microscope followed by quantification with ImageJ (Figures 2(b) and 2(c)) or flow cytometer (Figure 2(d)). The results showed that the intracellular ROS level increased significantly after TERT knockdown. The results also suggested that increased level of ROS may be a factor mediating cell growth inhibition by TERT deficiency. To confirm this notion, we treated TERT-deficient cells with the antioxidant NAC and used PBS as a control, and then the viability of the cells was detected. As expected, the cell viability was significantly restored after ROS removal, indicating that ROS was involved in the regulation of hTERT on cell viability (Figure 2(e)).

### 3.4. BECN1 Mediates the Effects of TERT on Autophagy, ROS Level, and Cell Survival in GBM

**Figure 3:**
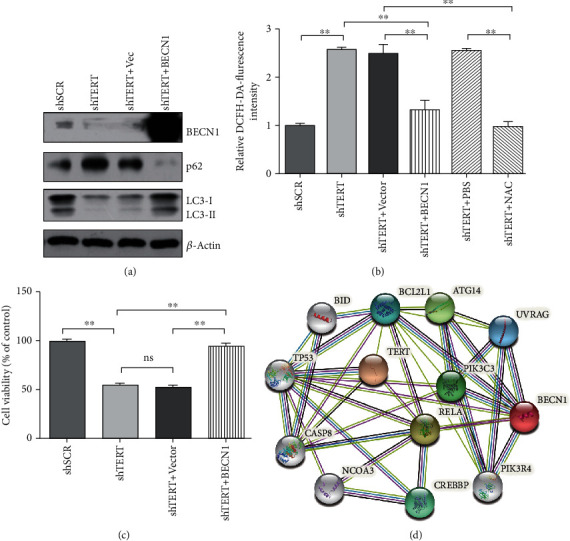
BECN1 mediates the effects of TERT on autophagy, ROS levels, and cell survival in GBM. (a) Western blotting images of autophagy-related proteins after overexpression of BECN1 in TERT knockdown cells. (b) Graphical representation of ROS levels in TERT knockdown cells overexpressed by BECN1 and treated with the antioxidant NAC. (c) Cell viability of TERT knockdown cells and control cells after overexpression of BECN1. ∗∗*p* < 0.01 and *p* < 0.05 were considered statistically significant. (d) Proteins in TERT-BECN1 interaction network searched by the STRING database.

BECN1 (Beclin-1) is a core mammalian autophagy protein necessary for the stage of vesicle nucleation. It binds to PI3K (phosphatidylinositol 3-kinase) to form a complex, which can recruit LC3 and initiate autophagy flux [26, 27]. To further investigate the mechanisms of TERT in regulating autophagy, we overexpressed BECN1 in TERT-deficient cells. As shown in Figure 3(a), autophagy could be restored by BECN1 overexpression. Accordingly, ROS level was also reduced after the recovery of autophagy (Figure 3(b)). These results indicated that BECN1 mediated the suppression of autophagy by TERT deficiency. To verify the effect of autophagy on the growth and proliferation of TERT-deficient cells, the viability of cells was detected. The result showed that the cell viability significantly decreased after stable knockdown of TERT protein. On this basis, the overexpression of the autophagy-related gene *BECN1* restored cell viability, indicating that BECN1-dependent autophagy was involved in the regulation of hTERT on cell growth and survival (Figure 3(c)). In order to further study the interaction relationship between TERT and BECN1 proteins, the STRING database was used to analyze their interaction protein network. There were eleven important proteins, ATG14, BCL2L1, BID, CREBBP, CASP8, NCOA3, PI3KC3, PI3KR4, RELA, TP53, and UVRAG that may play roles and were involved in the protein network of TERT and BECN1 (Figure 3(d) and Table 1).

## 4. Discussion

GBM has been considered the most common brain tumor with high recurrence and lethality [28]. The current option of medical therapy is relied on surgical resection followed by temozolomide treatment and/or radiotherapy [29, 30]. GBM patients usually have a poor quality of life and terrible prognosis after diagnosis. Thus, it is urgently needed to develop effective therapies. TERT has long been regarded as an important marker of cancer and a target of cancer treatment, but it has not been applied to clinical treatment. Previous studies showed that the vast majority of glioblastomas contain mutations in TERT promoter [31, 32]. In addition, the mutations of TERT promoter enhance its activity and serve as a mechanism for its regulation [10]. In this regard, we explored the expression levels of TERT mRNA in GBM samples by bioinformatic methods. Consistent with previous studies TERT expression is upregulated in a variety of tumor cells and its reactivation is a key step in the development of cancer [33–35]. Our results showed that the expression levels of TERT mRNA were higher in glioblastoma samples than that in healthy controls. Besides, the results of survival analysis showed that patients with high TERT expression lived shorter than those with low TERT expression. Biological experiments manifested that downregulation of TERT suppressed the growth and proliferation of U87 cells. These results indicated that TERT may promote the progression of GBM. It is well known that TERT is able to stabilize chromatin ends by lengthening telomeres. Moreover, TERT has other roles in mitochondria fitness regulation, antiapoptosis, and response to DNA break repair. Here, we discussed the relationship between TERT and autophagy. Our results showed that TERT deficiency impaired the process of autophagy mediated by BECN1 and led to a subsequently increased level of ROS. In addition, Overexpression of BECN1 or treating cells with antioxidant NAC decreased the levels of ROS and rescued the worse survival rate caused by TERT deficiency.

Autophagy is a cellular degradation process that maintains the balance of the intracellular environment, regulates cellular signals, and promotes cell survival. Promoting the survival of tumor cells under stress is widely recognized as an essential role of autophagy and makes it an attractive therapeutic target for cancer [18, 36]. Our results showed that TERT deficiency significantly inhibited the occurrence of autophagy and was depended on BECN1. The initiation step of autophagy is regulated by Class I and Class III PI3K. Class I PI3K can inhibit autophagy indirectly via mTOR, whereas Class III PI3K directly enhances autophagy through interaction with BECN1. Accordingly, the two pathways of mTOR and BECN1 may work together in autophagy [37, 38]. Our present study showed that TERT knockdown significantly decreased the expression of BECN1, the conversion of LC3B from LC3B-I to LC3B-II, and increase the expression of p62, indicating that TERT deficiency suppresses the process of autophagy in U87 cells. Ali et al. reported that TERT inhibits the kinase activity of mTOR complex 1 (mTORC1) in multiple cell lines, resulting in the activation of autophagy under both basal and amino acid-deprived conditions [39], whereas, in our present study, the expression level of p-mTOR did not significantly increase after TERT knockdown in U87 glioma cells. The change of BECN1 expression level was more significant than that of p-mTOR. Therefore, we speculated that BECN1 mediated the suppression of autophagy by TERT knockdown in GBM.

However, the mechanism of TERT regulating the expression of BECN1 needs further studies. A strong hypothesis is that hTERT indirectly regulates gene expression by acting as a transcriptional cofactor or via a posttranscriptional mechanism [40]. One mechanism comes from the interaction between TERT and a subunit of nuclear factor kappa B (NF-*κΒ*), suggesting that TERT responds to the regulation of NF-*κΒ* target genes, such as cytokines involved in cancer progression [17]. Moreover, another study found conserved binding sites of NF-*κ*B on the promoter of *BECN1* gene in mice and humans. The authors also showed that NF-*κ*B family member p65/RELA could upregulate the expression of BECN1 and promote the initiation of autophagy [41]. Therefore, it is highly possible that TERT regulates the expression of BECN1 by interacting with NF-*κΒ*. Besides, a study showed that TERT contains a BH3-like motif, a short peptide sequence found in BCL-2 family proteins. TERT interacted with MCL-1 and BCL-xL, antiapoptotic BCL-2 family proteins, suggesting a functional link between TERT and apoptosis pathway [42]. There are other studies reported that the antiapoptotic proteins BCL-2 and BCL-xL inhibited autophagy through their binding to the BH3-only protein BECN1 [43, 44]. Thus, BCL-2 family proteins might link TERT to BECN1 and was involved in the autophagic processes. In order to further study the interaction relationship between TERT and BECN1 protein, the STRING database was used to analyze their interaction protein network. We found that eleven proteins, ATG14, BCL2L1, BID, CREBBP, CASP8, NCOA3, PI3KC3, PI3KR4, RELA, TP53, and UVRAG, were involved in the protein network of TERT and BECN1. Our present results may provide a new idea for the mechanism of regulation of BECN1 by TERT.

Previous studies reported that TERT could function in response to oxidative stress and alleviate intracellular ROS levels [12, 45, 46], but the mechanisms are not well understood. It is suggested that an increase of cellular ROS can activate the process of autophagy, which will facilitate the removal of excessive ROS. On the contrary, when autophagy is impaired under certain conditions, ROS levels will be disturbed, which can affect the growth and proliferation of cells [47–49]. In the present study, we found that increased autophagy by BECN1 overexpression could lower the elevated ROS level in TERT knockdown cells. Based on these results, we speculated that BECN1-dependent autophagy may be a bridge builder mediating the regulation of ROS by TERT.

## 5. Conclusions

In summary, the present study provides insight into the roles of TERT on regulating ROS and autophagy during GBM progression. Our results demonstrate an interaction between TERT and autophagy mediated by BECN1. TERT deficiency impairs the process of BECN1-dependent autophagy, elevates the intracellular ROS level, and thus regulates cell survival and proliferation in GBM cells.

## Figures and Tables

**Table 1:** List of proteins in TERT-BECN1 interaction network.

Abbreviation of proteins	UniProtKB accession	Full names of proteins
ATG14	Q6ZNE5	Autophagy-related 14
BCL2L1	Q07817	Apoptosis regulator BCL-x
BID	P55957	BH3-interacting domain death agonist
CASP8	Q14790	Caspase 8, apoptosis-related cysteine peptidase
CREBBP	Q92793	CREB-binding protein
NCOA3	Q9Y6Q9	Nuclear receptor coactivator 3
PIK3C3	Q8NEB9	Phosphatidylinositol 3-kinase catalytic subunit type 3
PIK3R4	Q99570	Phosphoinositide-3-kinase regulatory subunit 4
RELA	Q04206	RELA protooncogene, NF-*κ*B subunit
TP53	P04637	Tumor protein P53
UVRAG	Q9P2Y5	UV radiation resistance-associated gene protein

## Data Availability

The data used to support the findings of this study are available from the corresponding authors upon request.
